# MicroRNAs in Preeclampsia: Bridging Diagnosis and Treatment

**DOI:** 10.3390/jcm14062003

**Published:** 2025-03-15

**Authors:** Angeliki Gerede, Sofoklis Stavros, Maria Danavasi, Anastasios Potiris, Efthalia Moustakli, Nikolaos Machairiotis, Athanasios Zikopoulos, Konstantinos Nikolettos, Peter Drakakis, Nikolaos Nikolettos, Makarios Eleftheriades, Ekaterini Domali

**Affiliations:** 1 Department of Obstetrics and Gynecology, Democritus University of Thrace, 691 00 Campus, Greece; mairidanavasi4800@gmail.com (M.D.); knikolet@med.duth.gr (K.N.); nnikolet@med.duth.gr (N.N.); 2 Third Department of Obstetrics and Gynecology, University General Hospital “ATTIKON”, Medical School, National and Kapodistrian University of Athens, 124 62 Athens, Greece; sfstavrou@med.uoa.gr (S.S.); nikolaosmachairiotis@gmail.com (N.M.); thanzik92@gmail.com (A.Z.); pdrakakis@med.uoa.gr (P.D.); 3 Laboratory of Medical Genetics, Faculty of Medicine, School of Health Sciences, University of Ioannina, 451 10 Ioannina, Greece; thaleia.moustakli@gmail.com; 4 Second Department of Obstetrics and Gynecology, University Hospital “Aretaieion”, Medical School, National and Kapodistrian University of Athens, 115 28 Athens, Greece; melefth@med.uoa.gr; 5 First Department of Obstetrics and Gynecology, Alexandra Hospital, Medical School, National and Kapodistrian University of Athens, 115 28 Athens, Greece; kdomali@yahoo.fr

**Keywords:** microRNAs (miRNAs), preeclampsia, gestational hypertensive disorders, biomarkers, therapeutic targets

## Abstract

Preeclampsia (PE) is a multifactorial hypertensive disorder that typically manifests after the twentieth week of pregnancy, significantly impacting perinatal mortality and neonatal morbidity. Its development is influenced by immunological components, systemic inflammation, and genetic factors, with placental malfunction playing a crucial role. While many aspects of its pathophysiology have been elucidated, its key mechanisms remain incompletely understood. MicroRNAs (miRNAs), small noncoding RNA molecules that regulate gene expression, have emerged as promising biomarkers and therapeutic targets in PE. Dysregulated miRNAs have been identified in pregnant PE patients, highlighting their role in disease onset. Placenta-specific miRNAs, such as miR-210 and miR-155, influence inflammation, endothelial function, and hypoxia responses, which are closely associated with PE development. These miRNAs play a crucial role in regulating trophoblast invasion, angiogenesis, and immune modulation, further linking their dysregulation to the pathophysiology of PE. This review aims to provide a comprehensive overview of the role of miRNAs in PE, focusing on their potential as diagnostic biomarkers and therapeutic targets. By integrating recent advancements in molecular research, we explore their implications in clinical practice, particularly in risk assessment, early detection, and novel treatment strategies.

## 1. Introduction

PE typically manifests after 20 weeks of pregnancy and is characterized by multi-organ damage. The hallmark symptoms include proteinuria, endothelial dysfunction, decreased extravillous cytotrophoblast invasion, hypertension, and restricted fetal growth. This severe condition contributes to approximately 70,000 maternal and 50,000 infant deaths annually, posing significant risks to both mother and fetus [1]. According to the 2020 NICE guidelines, PE is more likely to occur in women with a history of hypertensive disorders in previous pregnancies, as well as those with pre-existing conditions such as diabetes, autoimmune diseases, chronic renal disease, or chronic hypertension [2]. Other moderate risk factors include nulliparity, advanced maternal age (over 40 years), obesity (body mass index, BMI ≥ 35 kg/m^2^), multiple gestations, a family history of PE, and an interpregnancy interval longer than 10 years [3].

Emerging research indicates that some lifestyle and genetic factors may potentially contribute to PE susceptibility in addition to these well-established medical and demographic risk factors. Variations in PE risk, for example, have been linked to dietary practices, such as increased vegetable intake, alcohol consumption, and primigravidity [4]. Interestingly, a woman’s ABO blood group has been connected to pregnancy-related issues including PE [5]. These findings highlight the complex interplay of genetic, environmental, and lifestyle factors in PE development.

Despite significant advancements in our understanding of PE’s molecular underpinnings, many aspects of miRNAs in its pathophysiology remain unknown. Future studies should concentrate on miRNAs associated with ferroptosis, autophagy, and immunological responses because of their critical role in PE formation [6]. A deeper understanding of miRNA regulatory networks may improve our knowledge of PE pathophysiology and facilitate the identification of novel therapeutic targets. To validate these miRNAs and elucidate their functions in placental biology, functional studies are crucial [7].

MiRNAs were first discovered in C. elegans and later identified in humans as key post-transcriptional regulators. Advances in high-throughput sequencing and bioinformatics have led to the identification of numerous miRNAs associated with pregnancy and placental function. RNA sequencing and microarray technologies have been used to investigate the differential miRNA expression profiles in PE, providing insight into their potential for both diagnosis and treatment [8].

A more complete understanding of PE could be achieved by combining miRNA profiles with proteomic, metabolomic, and genomic data. Research into the complex interplay between genetic, epigenetic, and metabolic factors contributing to PE has proven feasible because of an increasing number of multi-omics techniques [9]. Precision medicine approaches to PE management may become possible as a result of the identification of new therapeutic targets brought about by such integrated research [10].

Cell-free DNA (cfDNA) has gained attention as a potential biomarker for the early detection of pregnancy complications, including PE. Recent research supports the use of cfDNA in precision medicine by showing that hypertensive diseases of pregnancy share common cfDNA methylation profiles [11]. Predictive models may be improved by combining cfDNA analysis and miRNA profiling, which would enable early risk assessment and customized management plans.

Currently, PE is diagnosed using biochemical markers and clinical symptoms, which usually do not present until much later in the course of the illness. Integrating miRNA profiles with proteomic, metabolomic, and genomic data could provide a more comprehensive insight into the molecular mechanisms driving PE. PE has well-established pathophysiological reasons, yet once the problem is recognized, therapy is still difficult. The majority of available treatments have adverse effects and symptoms. Early detection would significantly enhance management and reduce disease severity before clinical manifestations arise [12]. However, the lack of accurate diagnostic indicators and the poor association between severity criteria and unfavorable outcomes make early PE identification difficult. Finding trustworthy biomarkers has gained attention recently to support early detection and individualized treatment. Recent studies have demonstrated the critical role that miRNAs play in the development of PE, emphasizing their small size, stability, and abundance as critical characteristics that make them attractive targets for therapy and biomarkers. In contrast to earlier reviews that only address the therapeutic or diagnostic potential of miRNAs, this work incorporates both aspects and offers a thorough road map for their translational use in PE therapy [13].

This review explores the role of miRNAs in PE, focusing on their potential as biomarkers for early diagnosis and as targets for treatment. It consolidates current knowledge on their molecular mechanisms, examines their diagnostic and therapeutic potential, and discusses how miRNA-based approaches could be integrated into clinical practice. Additionally, it highlights existing challenges and outlines future research directions.

## 2. Molecular Mechanisms of miRNAs in PE

As PE progresses, anti-angiogenic molecules such as soluble endoglin and sFlt-1 are released, leading to systemic endothelial dysfunction and hypertension. Two key characteristics of the condition are placental insufficiency and poor vascular development, which are caused by these substances [14].

In addition to these anti-angiogenic compounds, miRNAs play a crucial role in regulating the pathological processes of PE. Angiogenesis is inhibited by miR-126, while miR-210 exacerbates hypoxic stress. These miRNA-driven mechanisms highlight the importance of further research to improve tailored PE therapies and early detection methods [15].

Oxidative stress and endothelial dysfunction play key roles in PE, disrupting vascular remodeling and repair [16]. A primary feature of PE is chronic hypoxia, leading to reactive oxygen species (ROS) accumulation, resulting in endothelial damage, trophoblast dysfunction, and inflammation [17].

Placental insufficiency is exacerbated by increased levels of miR-210 in PE, which inhibits trophoblast invasion and affects mitochondrial function. Additionally, miR-155 contributes to inflammation and endothelial dysfunction by downregulating the suppressor of cytokine signaling 1 (SOCS1), a negative regulator of pro-inflammatory signaling. However, miR-126 may have a dual role in PE, as its dysregulation not only affects angiogenesis but also impairs endothelial repair [18].

These findings highlight the potential of miRNAs as therapeutic targets for PE. By modulating the expression or activity of specific miRNAs, treatments could be developed to enhance endothelial function, reduce oxidative stress, and promote healthy placental vascular development. As research on miRNA-based therapies advances, more precise targeting of these pathways may lead to individualized and effective treatments for PE [19]. Ultimately, miRNA-based therapies hold promise for improving clinical outcomes and enabling more personalized care for PE [20].

## 3. MiRNAs as Diagnostic and Prognostic Biomarkers

The stability and disease-specific expression patterns of circulating miRNAs make them valuable biomarkers for PE. Some miRNAs facilitate early detection, as their expression levels change before symptoms appear. For instance, maternal plasma levels of miR-210, which is associated with placental hypoxia, rise significantly weeks before symptom onset. Another key biomarker, miR-21, enhances diagnostic utility due to its correlation with inflammation and placental dysfunction [21].

Reduced angiogenesis and endothelial dysfunction are linked to the downregulation of miRNAs such as miR-195 and miR-126. These distinct biomarkers highlight the potential of miRNA-based diagnostics for early risk assessment and intervention. Because miRNA-based assays provide non-invasive, early detection, they may be used in clinical practice to supplement current PE screening techniques. For instance, circulating miRNAs like miR-210 and miR-155 could be measured in maternal plasma using qRT-PCR or next-generation sequencing. In high-risk pregnancies, such methods could improve risk categorization and enable prompt interventions like aspirin prophylaxis [22].

Populations at high risk of PE, such as women with a history of PE, obesity, diabetes, autoimmune illnesses, chronic hypertension, and multiple gestations, may benefit most from miRNA testing. Further established risk factors where miRNA screening may be useful are first-time pregnancies and advanced maternal age (≥40 years). Because studies show that circulating miRNAs linked to PE, like miR-210 and miR-155, can be found before clinical symptoms manifest, the best time to perform miRNA testing would be in the first and early second trimesters (before 20 weeks of gestation). Early testing could improve risk stratification, which would also make preventative measures possible, like low-dose aspirin prophylaxis, which works best when started before 16 weeks [23].

Beyond screening, miRNA testing could also improve diagnostics by complementing existing biomarkers (e.g., the sFlt-1/PlGF ratio) to enhance predictive accuracy and differentiate PE from other hypertensive disorders of pregnancy. Furthermore, miRNA profiling may help classify disease severity, providing insight into the likelihood of adverse maternal and fetal outcomes [24].

Cost, consistency, and clinical validation are issues that miRNA-based diagnostics must deal with despite their promise. For routine obstetric care to be feasible, high-throughput, reasonably priced assays must be developed. Further studies are needed to assess the cost–benefit ratio of integrating miRNA profiling into existing prenatal screening programs [25].

A more useful and therapeutically relevant strategy might be offered by tailored panels that concentrate on important PE-associated miRNAs (such as miR-210, miR-155, and miR-126), even if thorough miRNA profiling may still yield insightful information [15].

The biochemical indicators (such as the sFlt-1/PlGF ratio) and clinical symptoms, which frequently manifest late in the course of the disease, are the mainstays of current PE identification. By enabling prompt interventions, miRNA-based testing may improve maternal–fetal outcomes by enabling earlier, more accurate risk assessment [26].

MiRNA profiles and clinical risk variables including maternal BMI and mean arterial pressure can be integrated into PE prediction models. Additionally, combining machine learning techniques with multi-omics data may enhance diagnostic accuracy and enable the development of individualized treatment strategies [27].

## 4. Therapeutic Potential of MiRNAs in PE

MiRNAs may significantly benefit PE as therapeutic agents due to their stability, specificity, and regulatory impact on key metabolic pathways. Their ability to alter gene expression at the post-transcriptional stage makes them ideal candidates for targeted therapy to reduce oxidative stress, placental dysfunction, and vascular abnormalities—key features of PE.

MiRNA inhibitors (antagomirs) and miRNA mimics are the two main approaches that have been suggested as miRNA-based treatments. Antagomirs work by inhibiting the activity of overexpressed miRNAs, like miR-210 and miR-155, to restore normal cellular processes. Alternatively, miRNA mimics improve placental development and vascular function by compensating for the loss of downregulated miRNAs likemiR-126 and miR-195 [21].

Crucial pathogenic processes in PE may be addressed by directly targeting these miRNAs, which may lower oxidative stress, inflammation, and endothelial dysfunction. Nevertheless, despite the therapeutic potential, there are still many obstacles to overcome before miRNA-based therapies may be used in clinical settings, especially when it comes to ensuring accurate and effective delivery to the placenta.

Developing targeted delivery systems is crucial because systemic injection poses risks of immune activation and off-target consequences. Exosome-mediated transport and lipid nanoparticles are two recent developments in nanotechnology that hold enormous promise for enhancing medicinal efficacy and delivery accuracy. Gene editing methods based on CRISPR may also provide a way to control the synthesis of miRNA in the future.

As research advances, clinical trials that go beyond preclinical validation in animal models will be crucial for assessing long-term effects, safety, and efficacy. Additionally, miRNA-based therapy may improve therapeutic results when combined with pharmacological treatments that are currently on the market. More targeted and effective treatments may also be possible with customized treatment plans based on miRNA profiling for high-risk pregnancies with PE [21].

### 4.1. miRNA-Based Therapeutic Strategies

Two main components of miRNA-based therapeutic interventions are miRNA mimics and antagomirs. Artificial analogs known as miRNA mimics account for downregulated miRNAs, while antagomirs prevent overexpressed miRNAs from causing pathological illnesses. The ability to resume their regular cellular functioning may be advantageous for PE patients using both approaches.

Therapeutic benefits may result from inhibiting miR-210, a key regulator of trophoblast invasion and mitochondrial function [15]. PE’s amplification of miR-210 degrades angiogenesis and increases oxidative stress, even if miR-210 aids mitochondria in better adapting to hypoxia. Accordingly, an miR-210 inhibitor may maintain mitochondrial function while reducing the adverse effects of mitochondrial malfunction [28].

Cytokine signaling-regulating miR-155 inhibitors could reduce inflammation and endothelial dysfunction. MiR-155 increases the production of TNF-α and IL-6 and fosters a pro-inflammatory environment in PE via targeting cytokine signaling suppressors (SOCS1 and SOCS3). Blocking miR-155 may therefore improve vascular health and reduce systemic inflammation [29]. To regulate angiogenesis, MiR-125b modifies extracellular matrix remodeling, making it another interesting target. MiR-125b mimics may improve placental vascularization and encourage healthy trophoblast function, which could help resolve the vascular inadequacies observed in PE [30].

### 4.2. Delivery Challenges, Solutions, and Future Directions

Effective distribution of miRNAs is hampered by their inability to target the placenta, achieve specificity, and guarantee safety, despite their therapeutic potential. Systemic administration of miRNA-based therapeutics carries the potential of off-target effects and degradation prior to placental distribution. Several innovative distribution techniques are being researched to address these problems [31].

Nanotechnology has facilitated the development of lipid nanoparticles (LNPs), polymeric nanoparticles, and exosome-based carriers for customized miRNA distribution. Exosome-based carriers enhance placental targeting and biocompatibility, while LNPs inhibit miRNA degradation, encourage cellular absorption, and permit controlled release. Researchers are also investigating peptide-functionalized nanoparticles for targeted trophoblast distribution and placental exosome-mimicking vesicles to lessen systemic exposure and adverse consequences. CRISPR interference (CRISPRi), a gene-editing technique, is another innovative tactic that may allow for precise regulation of miRNA expression and offer a possible permanent solution for dysregulated miRNAs. However, more research is required because gene editing during pregnancy presents ethical and safety concerns [32].

Thorough preclinical and clinical research is necessary to assess the long-term effects, safety, and efficacy of miRNA-based therapies before they are used in clinical settings. Gene knockout mouse models and placental ischemia-induced rat models are two well-established PE models that can be used to evaluate the efficacy of miRNA-targeted treatments. The best doses, biodistribution, and off-target dangers can all be ascertained using these models. Furthermore, traditional PE therapy could be combined with miRNA-based therapies. For instance, miR-126 mimics and antihypertensives could decrease blood pressure fluctuations and encourage vascular healing [15].

Additionally, prophylactic miRNA therapy may help high-risk women (such those with a history of PE) before symptoms manifest. Timely therapy with miRNA mimics or inhibitors may be made possible by early biomarker screening for dysregulated miRNAs (such as miR-210 overexpression). Despite their current experimental stage, miRNA-based therapies hold promise for future treatment approaches due to their capacity to target particular biochemical pathways in PE. Further developments in gene editing, delivery technologies, and clinical validation are required to improve maternal and fetal outcomes in PE and move miRNA-based therapy closer to clinical implementation [33]. This review goes beyond conventional miRNA-based therapies by exploring CRISPR-mediated miRNA regulation and advanced delivery mechanisms, paving the way for highly specific, long-lasting interventions in PE.

### 4.3. Future Directions in Therapeutic Development

MiRNA-based medications must pass rigorous preclinical and clinical testing to assess their long-term effects, safety, and effectiveness before being used in clinical settings. In each animal model of PE, for instance, the optimal dosing schedules for potential miRNA-based therapeutic strategies can be investigated. Additionally, there may be some advantages to combination therapy, which combines miRNA-based strategies with other traditional treatments such antibiotics or antihypertensive medications. Treatment with protective miRNA mimics or inhibitors may help lower the severity of the disease’s development in high-risk groups as well. For example, women who have previously suffered from diseases like PE may benefit from using miRNAs in preventative treatments [34,35].

## 5. Dysregulated MiRNAs in PE

Systemic inflammation, abnormal placental development, and endothelial dysfunction are the hallmarks of PE, a hypertensive syndrome linked to pregnancy. Dysregulated miRNAs that impact trophoblast invasion, angiogenesis, immune response, and oxidative stress are increasingly implicated in the pathophysiology of PE. Numerous investigations have revealed that maternal blood and preeclamptic placentas have either elevated or downregulated levels of certain miRNAs [16].

MiRNA-210 is among the most upregulated because it targets vascular endothelial growth factor (VEGF) and hypoxia-inducible factor (HIF-1α) to prevent trophoblast invasion and angiogenesis. Similarly, miR-155, which modulates SOCS1 and endothelial nitric oxide synthase (eNOS) and is associated with endothelial dysfunction and elevated inflammation, is found at higher levels in preeclamptic placentas. Another upregulated miRNA, miR-21, targets phosphatase and tensin homolog (PTEN) and programmed cell death 4 (PDCD4) to influence apoptosis and immune response [36,37].

Placental function is diminished in PE, however, as a result of many miRNAs being downregulated. Vascular dysfunction results from preeclamptic individuals’ markedly reduced levels of miR-126, which targets VEGF-A and insulin-like growth factor (IGF-1) to stimulate endothelial cell proliferation and angiogenesis. The downregulation of miR-195, which regulates B-cell leukemia/lymphoma 2 protein (BCL2) and wingless-related integration site (Wnt) signaling to promote trophoblast proliferation and survival, exacerbates placental insufficiency [38].

Increased inflammation, poor angiogenesis, elevated oxidative stress, and impaired trophoblast invasion are some of the main pathogenic characteristics of PE that are influenced by the dysregulation of these miRNAs. Inadequate vascular remodeling results from reduced production of pro-angiogenic miRNAs such as miR-126, whereas trophoblast activity and oxygen balance are compromised by overexpression of miR-210. Elevated levels of miR-155 lead to excessive inflammation, which worsens maternal endothelial dysfunction. The findings suggest that miRNAs coordinate critical cellular processes and play a multifaceted role in the development of PE [30].

Circulating miRNAs have demonstrated potential as biomarkers for the early diagnosis of PE in addition to their function in the pathogenesis of disease. Research has shown that reduced levels of miR-126 and miR-195 are associated with vascular dysfunction and placental hypoxia, while maternal plasma levels of miR-210, miR-155, and miR-21 are enhanced weeks before the onset of clinical symptoms. MiRNAs are appealing candidates for non-invasive diagnostic testing because of their stability in the bloodstream and their expression patterns specific to particular diseases. The potential of these markers to direct clinical therapy must be investigated further, and their validity in broader patient populations must be confirmed [21].

Novel therapeutic approaches and insights into the mechanisms behind PE are made possible by an understanding of the role of miRNAs in the disease. Restoring normal placental function and improving pregnancy outcomes may be possible by using miRNA mimics or inhibitors to target dysregulated miRNAs [39]. Table 1 summarizes the dysregulated miRNAs in the placentas and maternal blood circulation of preeclamptic patients.

## 6. Future Research Directions

### 6.1. Discovery and Mechanistic Studies of Novel miRNAs

Despite significant progress, many miRNAs involved in PE remain unidentified. Future research should focus on discovering novel miRNAs and elucidating their mechanistic roles in disease progression. High-throughput RNA sequencing and bioinformatics approaches can aid in identifying new miRNAs that regulate vital biological processes such as immune response, ferroptosis, and angiogenesis.

To validate these regulatory functions, functional studies using trophoblast and endothelial cell models should be conducted. Advanced techniques, including miRNA overexpression/inhibition and clustered regularly interspaced short palindromic repeats (CRISPR/Cas9) gene editing, can help clarify their roles in trophoblast invasion, vascular remodeling, and oxidative stress responses. Additionally, in vivo validation through genetically modified animal models will be essential to uncover the systemic effects of dysregulated miRNAs in PE [40,41].

### 6.2. Integration with Multi-Omics Approaches

Multi-omics approaches including proteomics, metabolomics, and genomics, should be integrated with miRNA profiling to achieve a more thorough understanding of PE. These advanced techniques enable the exploration of complex interactions among metabolic, genetic, and epigenetic factors that contribute to PE pathogenesis. For example, combining miRNA profiles with proteomic data on angiogenic factors could improve prediction models for early PE detection. Furthermore, multi-omics research may not only improve disease comprehension but also facilitate the identification of novel therapeutic targets, paving the way for personalized PE treatments [42,43].

### 6.3. Development of Predictive Models

It is essential to develop prediction models that incorporate miRNA profiles for the early detection and risk classification of PE. This need arises from the clinical challenges associated with PE diagnosis, which often relies on delayed biomarkers and subjective symptoms. Combining clinical parameters such as BMI and mean arterial pressure with miRNA levels (e.g., miR-210 and miR-155) could significantly enhance diagnostic accuracy. Furthermore, machine learning algorithms can analyze multi-omics data to generate robust, personalized predictive models, enabling timely intervention, particularly in high-risk pregnancies [15,39]. Pregnancies at risk can benefit from tailored treatment plans and accurate risk classification made possible by utilizing these cutting-edge computational tools [44].

### 6.4. Long-Term Vision for miRNA Research in PE

Ultimately, the goal is to translate miRNA discoveries into precision medicine strategies for PE, allowing for early identification of high-risk pregnancies and personalized therapeutic interventions. Maternal and fetal health outcomes can be greatly improved by combining multi-omics data, developing pharmaceutical advances, and enhancing miRNA-based diagnostics. MiRNA-driven approaches should play a central role in PE prevention and therapy. Future research must continue bridging the gap between experimental findings and clinical applications to ensure these strategies are effectively implemented (Table 2) [45].

## 7. Discussion

This study highlights the critical roles of many miRNAs in the pathophysiology of PE, particularly in vital processes such as trophoblast invasion, extracellular matrix remodeling, inflammation, and angiogenesis. This review underscores the potential of miRNA-based precision medicine in PE, emphasizing the integration of miRNA profiling with multi-omics approaches to enable individualized risk assessment and targeted therapeutic strategies. Dysregulated miRNAs, including miR-31-5p, miR-155-5p, miR-125b-5p, miR-210-3p, and miR-218-5p, are associated with placental failure, the hallmark of PE. Given their potential as biomarkers and therapeutic targets, further studies are needed to validate their clinical applications.

Currently, miRNA-based assays are not yet widely integrated into routine clinical practice but are being actively researched for their diagnostic potential in PE. In clinical practice, miRNA profiling could complement existing PE screening methods by offering non-invasive, early detection. Circulating miRNAs such as miR-210 and miR-155 can be measured in maternal plasma using qRT-PCR or next-generation sequencing, enhancing risk stratification and enabling timely interventions such as aspirin prophylaxis in high-risk pregnancies. However, despite their promise, miRNA-based diagnostics face challenges related to cost, standardization, and clinical validation. The development of cost-effective, high-throughput assays is essential to ensure feasibility in routine obstetric care. As of now, the integration of miRNA profiling into prenatal screening programs remains an area of active research, and further studies are needed to assess the cost–benefit ratio before clinical adoption [13,46].

While comprehensive miRNA profiling provides valuable insights, a targeted approach focusing on key PE-associated miRNAs (e.g., miR-210, miR-155, miR-126) may offer greater clinical utility. Current PE detection methods rely on clinical symptoms and biochemical markers such as the sFlt-1/PlGF ratio, which often become apparent only in later disease stages. In contrast, miRNA-based tests could enable earlier, more accurate risk assessment, potentially improving maternal–fetal outcomes by facilitating timely interventions. Unlike conventional biochemical markers, miRNAs offer a dynamic and early reflection of pathological changes in PE, making them a promising alternative in predictive models [13].

Non-invasive screening of maternal plasma presents a promising diagnostic approach due to the early dysregulation of miRNAs in PE. MiRNAs associated with hypoxia, oxidative stress, and inflammation could serve as therapeutic targets for novel treatment strategies. To optimize miRNA-based therapies, future research should focus on refining targeted delivery methods and integrating miRNA signatures into predictive models for improved risk assessment and intervention [47,48].

MiRNA-based diagnostics and therapeutics represent a promising frontier in PE management. Their integration into clinical practice could enhance early detection, risk stratification, and personalized treatment strategies. However, challenges related to standardization, targeted delivery, and cost-effectiveness must be addressed before widespread clinical adoption [48].

**Figure 1:**
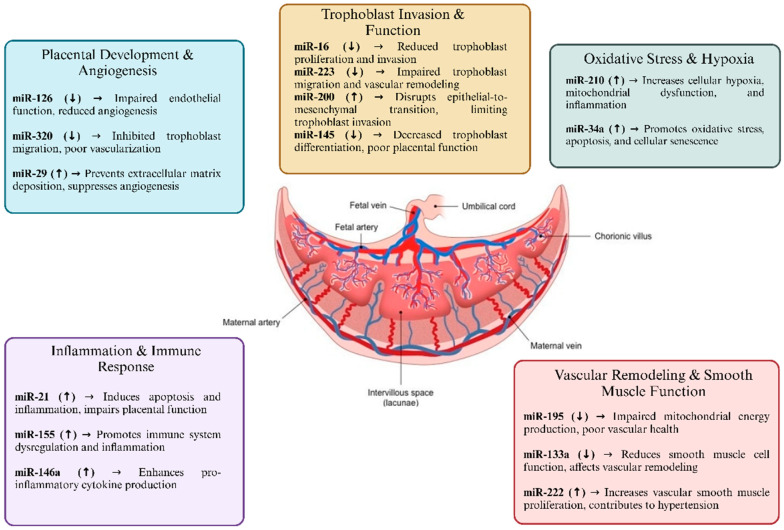
The functions of several miRNAs in the pathophysiology of PE are depicted in this picture. The main roles of miRNAs are divided into several categories, such as trophoblast invasion, inflammation and immunological response, oxidative stress, vascular remodeling, and angiogenesis regulation.

Figure 1 depicts the functions of several miRNAs in the pathophysiology of PE and Table 3 summarizes all the included miRNAs in this review with details of their function and role in PE.

## 8. Conclusions

MiRNAs play a pivotal role in the pathophysiology of PE and offer great potential as both diagnostic markers and therapeutic targets. Their ability to regulate key cellular processes and metabolic pathways makes them promising strategies for addressing the underlying mechanisms of PE. However, translating these encouraging findings into clinical practice remains challenging. Reducing off-target effects and guaranteeing the accurate and focused distribution of miRNAs to the placenta are major challenges.

To overcome these challenges, future research must focus on developing safer, more effective delivery systems. Enhancing prediction models that incorporate miRNA profiles will also be essential for increasing diagnostic skills; this may be accomplished through multi-omics techniques. Integrating miRNA-based therapies with existing pharmaceutical treatments offers a pathway to personalized and more effective treatment strategies for PE.

Through improved early diagnosis, risk assessment, and individualized treatment, miRNA-based diagnostics and treatments could revolutionize PE management as research advances. However, issues including cost-effectiveness, delivery methods, and standardization must be resolved before broad clinical adoption can occur. By identifying new therapy targets and making it easier to find more precise biomarkers, the combination of miRNA profiling and multi-omics approaches may pave the way for new developments in PE research. Ultimately, these developments greatly enhance outcomes for mothers and infants, representing a major advancement in the management and treatment of PE.

## Figures and Tables

**Table 1:** Dysregulated miRNAs in the placentas and maternal blood circulation of preeclamptic patients.

miRNA	Expression in PE	Target Pathway	Function
miR-210	Upregulated	HIF-1a, VEGF	Inhibits trophoblast invasion and angiogenesis
miR-155	Upregulated	SOCS1, eNOS	Promotes inflammation, endothelial dysfunction
miR-21	Upregulated	PTEN, PDCD4	Regulates immune response and apoptosis
miR-126	Downregulated	VEGFA, IGF-1	Impairs endothelial cell proliferation and angiogenesis
miR-195	Downregulated	BCL2, Wnt signaling	Reduces trophoblast proliferation

**Table 2:** Main areas of study for miRNA-related PE. In order to ensure optimal PE management, it encompasses the following topics: discovery, which focuses on finding novel miRNAs; biomarker development, which aims for early detection; therapeutics, which explores miRNA-based treatments; and clinical translation.

Research Area	Key Focus	Proposed Approaches
Discovery and Mechanistic studies	Identify novel miRNAs and validate their roles	High-throughput sequencing, CRISPR/Cas9, functional assays, animal models
Translational Biomarker Development	Establish miRNAs as early diagnostic markers	AI-powered predictive models, multi-omics integration, and longitudinal cohort studies
Therapeutic Development	Develop miRNA-based treatments for PE	miRNA mimics/inhibitors, combination therapies, targeted delivery systems (LNPs, exosomes)
Clinical Translation	Manage PE by implementing miRNA-based therapies.	Personalized medicine tactics, clinical trials, and preclinical validation

**Table 3:** List of all included miRNAs in the review with details of their function and role in PE.

miRNA	Role in PE	Function
miR-210	Associated with placental hypoxia, poor vascular remodeling, and compromised trophoblast invasion.	Promotes inflammation, hypoxia, and cellular dysfunction.
miR-29	Prevents the processes of extracellular matrix deposition and angiogenesis, which are critical for placental development, from occurring.	Impacts extracellular matrix deposition and angiogenesis.
miR-21	Impairs placental function and induces systemic inflammation.	Regulates apoptosis and inflammation.
miR-195	Downregulated; linked to impaired mitochondrial energy production and vascular development.	Controls mitochondrial activity and vascular health.
miR-126	Reduced angiogenesis and impaired endothelial function are linked to decreased expression in PE.	Regulates angiogenesis and endothelial cell function.
miR-155	Increases during PE, linked to changes in the immune system and inflammation.	Causes immune system dysfunction and inflammation.
miR-16	Downregulated in PE, associated with poor placental development and decreased trophoblast invasion.	Controls trophoblast activity, apoptosis, and the cell cycle.
miR-124	Reduced PE, linked to vascular remodeling and endothelial dysfunction.	Regulates endothelial function and vascular balance.
miR-152	Gene expression variations and placental abnormalities are associated with downregulated PE.	Impacts gene expression and DNA methylation.
	Participates in angiogenesis and placental development; decreased in PE.	Promotes trophoblast invasion and angiogenesis.
miR-34a	PE increases are linked to cellular aging and oxidative damage.	Modulates cellular aging, oxidative stress, and apoptosis.
miR-146a	Immune dysregulation and inflammatory processes are linked to upregulated expression in PE.	Interferes with inflammatory signals and the immunological response
miR-223	Downregulated in PE, associated with vascular remodeling and trophoblast invasion.	Maintains vascular homeostasis and controls trophoblast activity.
miR-200	Trophoblast invasion and the epithelial-to-mesenchymal transition are both compromised in PE.	Controls the migration, invasion, and transition of cells from epithelial to mesenchymal.
miR-145	Placental function and trophoblast development are both impacted by decreased PE.	Influences placental growth and trophoblast differentiation.
miR-133a	Downregulated in PE, associated with vascular remodeling and smooth muscle cell proliferation.	Affects vascular tone and the activity of smooth muscle cells.
miR-222	Increased in PE, implicated in vascular smooth muscle cell proliferation and endothelial dysfunction	Modulates the behavior of vascular smooth muscle cells and endothelial function.
miR-195	Downregulated; associated with compromised vascular development and mitochondrial energy generation.	Regulates vascular health and mitochondrial activity.
